# The Forces of Organic Life—with Some Remarks on Dr. Breed’s Case of Ileitis*Chicago Medical Examiner, for October, 1869.

**Published:** 1869-12

**Authors:** Z. C. McElroy

**Affiliations:** President Muskingum County Medical Society, Zanesville, Ohio


### CHICAGO MEDICAL EXAMINER.

N. S. DAVIS, M.D., Editor.

VOL. X. DECEMBER, 1869. NO. 12.

### Original Contributions.

### ARTICLE XLII.

### THE FORCES OF ORGANIC LIFE—WITH SOME REMARKS ON DR. BREED'S CASE OF ILEITIS.*

By Z. C. McELROY, M.D., President Muskingum County Medical Society, Zanesville, Ohio.

The case described by Dr. Breed is an extremely interesting one, as is also his manner of reporting it. In commenting on the facts, as he has presented them, it is not my purpose to censure him, but to present an analysis of its phenomena, from a different standpoint, and conclusions somewhat different from those at which he arrived; for, it seems to me, he failed to comprehend it, either before or after death, though he scrutinized it so closely. Working in the accredited methods of investigation, his conclusions are fully warranted by the philosophy by which he was guided. The purpose of this paper is to review it from a physical basis of life, in the same way as any other problem in physical science would be studied, on its facts alone.

For my purposes, he does not report what to me would be the most important single item in the daily record of his patient's condition—his temperature, ascertained by a correct thermometer in the axilla. The fact of his patient being a "compositor,"

* Chicago Medical Examiner, for October, 1869.

seems not to have directed his attention to the possibility of lead having something to do with his patient's condition and death. His *post mortem* would have been more complete had he extended his examination to the gray matter of the nerve plexuses, and lower portion of the spinal chord.

The following are, briefly, the principles of organic life, on a physical basis, which will guide me in the study of Dr. B.'s case:—

1st. The reign of law, in organic and inorganic nature, is supreme and invariable. The order, harmony, and perpetuity of the universe depend upon the absolute inflexibility of natural law. None flinched or failed in Dr. B.'s case.

2d. The total quantity of matter and force in the world is fixed and unchangeable. Nothing can be added and nothing destroyed. The forces of the inorganic world are mutually convertible into each other, and each into all; and the forces of organic life are correlated with the physical forces. There is, in fact, an absolute unity of force, in organic and inorganic nature.

3d. Excluding from the human body known inorganic elements and the operation of the known physical forces of light, heat, electricity, magnetism, etc., and there is left nothing but its *forms*, *i.e.*, the types or shapes of the tissues, textures, and organs—and that is all there is of the hitherto mysterious and incomprehensible vital force, about or in it. Vitality, therefore, is the preserver of form, with the momentarily changing material of its tissues and textures. Over forms, normal or abnormal, therapeutic agents have no control or influence whatever, except to destroy; therefore, as physicians, we need not trouble ourselves about vitality, in our ministrations to the sick. All that we can influence by therapeutic, remedial, or hygienic agencies is, the matter and force of the inorganic world, organized as food by the vegetable kingdom; or the vegetable matter in organized tissues of other animals, in its ascent, through the processes of digestion and assimilation, to the normal types and forms of organic life, with normal dynamic capabilities; and the decay, oxidation, or waste of the tissues in the evolution of normal organic dynamics, as heat, mechanical motion, sensation, emotion, chemical affinity, and intellection.

4th. A conception of the human body, embracing most of its prominent facts, in the evolution of force, is obtained in the study of a burning candle, which gives light and heat, because it, the candle, is being consumed. So, of organic dynamics, each result, be it thermal, mechanical, chemical, sensory, emotional, or intellectual, is at the expense of the tissues; thus, so much heat, so much tissue resolved into carbonic acid, water, and urea, or other products evolved in the decay of organic tissues; so of mechanical, chemical, sensory, emotional, and intellectual results, so much tissue resolved into carbonic acid, water, and urea. Thus, this very identical memoir, whatever it may be worth to those who may choose to read it, has been composed at the expense of the gray, brain matter and muscles, and other tissues of the author; and so of the reader, for every impression received by his mind, so much brain and other tissue reduced in organization, and started on its way back to the positions of its elements in inorganic nature.

The thermometer measures the rate of tissue waste—nothing more, nothing less—in accordance with the law, "for every dynamic result there must be change of matter," whether in organic or inorganic natures; and that is the impassable gulf in the way of a "perpetual motion" machine; and hence the importance of the thermometer, clinically, for a single degree of variation from the normal temperature of the body marks too much or too little waste. To maintain organic life in its highest perfection, each day's waste must be repaired, and each day's repair wasted.

5th. The human body is a unity, an integer, composed of many fractions, in arranged and disarranged conditions, *i.e.*, in health and disease. Whatever impressions are made or received on one part are instantly communicated, through the white matter of the nervous chords, to the whole organism. There is, therefore, no such a thing as a local disease—all the nosology of the past and present to the contrary notwithstanding.

6th. The influence, or action, or effects of all therapeutic, remedial, and hygienic agents, or measures, are general, *i.e.*, influence the whole body, and not a part of it, as the nomenclature of the *materia medica* and nosological classifications of diseases teach and imply.

7th. The disease is always something less, not something added, to normal life. The human tissues in health are examples of the highest organization in the universe, are the very perfection of organic life, and all changes are to lower states of organization, and to death.

8th. That the patient is, under all circumstances whatever, superior to his or her maladies, and should be the main object of care on the part of the physician. Disease, or a disarrangement of the body, is always of secondary importance to the patient. Homeopathy, hydropathy, eclecticism, and other empirical systems of practical medicine are gigantic protests against, as well as grotesque caricatures of, the philosophy and practice of regular medicine. What regular medicine needs is a central idea or theory of life, embodying its empirical facts and experiences, in health and disease. Practitioners of medicine rarely rise above their philosophy. Physical science can supply this need.

9th. The nerve masses, containing gray and white matter, are the organic electro-magnetic batteries, evolving organic force. The nerve chords, containing only white matter, are, like the telegraph wires, which now so nearly girdle the earth, conductors of force only, or, perhaps, more properly, force and impressions. The larger and smaller brain, medulla oblongata, and spinal chord are the main force evolving masses. The ganglions and plexuses, containing gray matter—the so-called sympathetic system—one supplemental to the main masses, and are found only where important organs are to be presided over, so as to render the performance of their allotted functions, or offices, to some extent, independent of the main masses. The necessity for this anatomical arrangement is obvious enough; for, were it otherwise, concussions or shocks from falls, accidents, and moral impressions would more uniformly prove fatal to life; for these act by suspending destructive metamorphosis, or the changes of matter evolving force; and as life, or organic dynamics, depend absolutely on changes of matter, the result, very frequently, would be a true enthanasia.

The facts stated by Dr. Breed, in relation to his case of ileitis, are as follows:—

*1st day.* The patient, a lad, 16 years of age, a compositor, after a chill, had some fever; pulse 80, moderately full and strong; pain in the bowels, somewhat paroxysmal, though never quite easy. The bowels were a little distended, elastic, and a little harder to the feel than natural, with tenderness on pressure. Tongue moist and covered with a dark, slimy coating. Had had no motion in bowels for 24 hours. Had vomited freely. The treatment instituted by Dr. B. is not noticed, as it falls outside of the purposes of this memoir.

*2d day.* No motion from bowels; urine free, but not remarkably high-colored. Vomiting persistent, decidedly bilious, and very dark; pulse 98, soft, rapid, and undulating; artery large; circulation languid; copious perspiration during sleep; tenderness of abdomen much the same; not much distension, though uniform; no flatus, or meteorismus; on applying ear to the abdomen no sound was heard, no gurgling—a dead silence prevailed throughout the abdominal region; during vomiting, muscles of abdomen did not coöperate with those of thorax; matter vomited, grass green, and acid.

*3d day.* Bowels not moved; vomiting continues; countenance haggard; takes no nourishment, except beef-tea, in enemata.

*4th day.* Less sweating than third day; vomited occasionally; countenance anxious; very restless; passed urine several times; pulse 100; artery large, felt soft and undulating under the finger, against which the pulse dashed with wave-like impression, and was gone; prostration evidently increasing. In the afternoon, pulse 100; passed some fecal matter; vomiting very distressing; hiccough.

*5th day.* Vomiting continues; small fecal discharges.

*6th day.* Vomiting; has become stercoraceous; passed urine several times; pulse 120, soft and undulating; countenance hippocratic; restless; somewhat delirious; no meteorism; bowels slightly tympanitic; death, soon after midnight.

It is further remarked (p. 584), there was no disposition to flex the legs on the abdomen; could lie on either side.

*Post Mortem.*—Abdomen moderately distended; peritoneum thickened, softened, its bloodvessels enlarged, very dark color, but no effusion; small intestines nearly empty, not collapsed, but generally discolored, with extensive adhesions in various places; muscular and mucous coats of intestines dark, thickened, nearly in a state of sphacelus; stomach not opened, but its external appearance seemed healthy; gall-bladder moderately distended with healthy bile; other organs examined were healthy; no mechanical obstruction; no impacted feces, or undigested matter found in bowels.

The facts of symptomology are only reproduced, and mainly in the very words of Dr. B.; though, for brevity, some sentences have been slightly abridged, but not so as to impair or alter the sense in which he used them.

A study of this young man's case, in the light of the general principles laid down, the phenomena which so sorely puzzled Dr. B. become less opaque and more understandable.

The patient was a compositor, handling leaden types during working hours; perhaps, like other compositors, holding type in his mouth very often, which is, probably, the clue to unravel the whole case. On the first day, symptoms, a negation; was probably confined to the house, possibly to bed. No mention is made of the duration of the prodromic symptoms beyond a few hours. He is weak, has some pain in the bowels, and has vomited. The absence of the temperature of his body is a very serious gap in the detail of the symptoms. It may have been above or below the normal standard; but, most likely, would have shown a range of two or more degrees above, exactly corresponding with the chemical changes taking place in the abdomen; for these chemical changes would, most probably, be correlated in heat. The state of the temperature would have given the key to the proper treatment. Here, the question as to whether it was the patient himself, or only his bowels that were sick, would, with me, have naturally come up for settlement and decision. The thermometer would have greatly aided me, for if the temperature was much above natural, then it would have been clear to me that it was the patient himself that was sick; and as all patients are superior to their maladies, the patient would have been the principal object of professional care. And by this is meant, that no *coup de etats* would have been undertaken to catch the disease off its guard, and thus kill or expel it, and so cure the patient at once. But supposing that it was the dreaded *inflammation* that was in progress in the .patient's abdomen, as shown by the increase of temperature, the question, what is inflammation? might be appropriately discussed in the mind. Is it a higher or lower grade of organic life than health? Is it something added to or substracted from the normal condition of the tissues in which it may be located? Does it tend to a condition higher than life, and, therefore, desirable? or does it tend to death? If it tends to death, what is it? Is it like a viper, a something foreign to the body, which has gained access to its interior? Or, is it a super-oxidation of tissue, not yielding normal organic force? Is the tissue thus wasted being replaced? Are the processes of repair and waste normal? These may be easily answered. Inflammation is a super-oxidation of tissue, with a suspension of repair in normal type, the chemical changes being correlated as heat, and not normal organic force. It is nothing more, nothing less, than a natural process taking place too rapidly, with repair arrested; the blood plasma, failing to reach the normal type of tissue, is poured out into the meshes of the wasting tissue, or on its surfaces, constituting the thickening spoken of by Dr. B. The decay of this exudation constitutes pus.

But the case was a negation, the first day. Second day, the ear detects no sounds in the abdomen; the legs are not drawn up; the pulse is feeble; there is vomiting. What is the significance of these facts? Failing power is their lesson. Where? The sources of power are the nerve masses, containing gray matter, and it is to them attention must be turned for an explanation. Nothing is said of the state of the pupil by Dr.

Breed, at any time. I do not know why this circular muscle of the eye should be so called. While its main purpose is, undoubtedly, to regulate the admission of light into the posterior chambers of the eye—expanding, as it does, in dim, and contracting in strong or bright, light—it incidentally serves another and still more important purpose, to those who will observe it, in revealing the condition of the forces of organic life. It is, so to speak, the dial on which is momentarily recorded the condition of the forces of life. In Dr. Breed's case, it was probably widely expanded and sluggish, and, if so, it was additional evidence that the phenomena detailed by Dr. Breed indicated failing organic power.

How and why did this young man's dynamics fail? He was a "compositor," handling type, mainly composed of lead, during all his working hours; and the probabilities are that the failure of the dynamics was due to lead poisoning, or lead paralysis.

What is lead poisoning? What is its *modus operandi* in producing colic and paralysis?

Referring again to the burning candle, as affording the best conception of human organic dynamics—though equally applicable to all animal organic dynamics—how would incombustible matter, as lead, combined with its tallow, be likely to influence its combustion? A candle requires a soft, spongy wick. When flame is first applied to the wick, in a new candle, it burns very feebly for a time. After a while, a portion of the end of the wick becomes charred, but still very porous, and around it, at a certain distance below the flame, a little cup is formed, filled with tallow, rendered fluid by the heat from the combustion going on above it. This fluid tallow is drawn up through the soft, spongy wick to the junction of the charred and uncharred wick, where it is decomposed or consumed; the tallow resolved into light, heat, aqueous vapor, and carbonic acid. The charred portion of the wick, in the heat of the flame, soon solidifies, so that fluid tallow cannot pass through it, and be divided, so that combustion can take place. This it is requisite to remove, to obtain the maximum of light. Now, suppose minute portions of oxide of lead were diffused through the tallow, it would fill up the spongy portion of the wick much more rapidly; and if it were snuffed all the time, it would still give much less light than it would if the tallow contained no incombustible matter, or matter incapable of assuming the gaseous or liquid forms. This gives me a clear mental conception of lead poisoning and lead paralysis. The lead may be interslitially deposited in the constructing tissues, or it may otherwise act by preventing the construction of tissue in normal type, with normal dynamic capabilities. Perhaps it may act in both ways. But it must act in one or the other, and it detracts nothing from the value of the conception which, or in both. As it cannot be made to assume either the fluid or gaseous form, by the chemistry of organic life, it accumulates in the tissues, and interferes with their proper oxidation, and the evolution of normal organic force; for only normal tissue can evolve normal force by its decay. And that, it seems probable, is all there is, or can be, of lead poisoning, or lead paralysis.

It may have been so in the lad treated by Dr. Breed, or it may not. But there certainly was a defective evolution of force, and that defect extended to the nerve masses containing gray matter; for, if they had been in a state of integrity, there would have been more pain, probably amounting to spasm. The problem of organic life for our study in the human body is, the conversion of food into tissue, and its oxidation, so as to produce normal dynamic force. In the young lad's case, tissue-making was certainly suspended, probably on the first day he was seen by Dr. B., but certainly on the second, and forever after. Oxidation, in the production of normal force, was as certainly suspended from, and, probably, prior to, the first day's treatment, though his candle did not cease to burn till the close of the sixth day. The *post mortem* revealed that the contents of the abdominal cavity had been partially surrendered to the process of ordinary putrefactive decomposition, some days before his dissolution. The nerve masses would, probably, have shown equally, or, perhaps, more striking changes, to the microscopically aided eye, and, very likely, to the unass'sted vision.

In studying the case, the leading fact to be borne in mind is, that all the phenomena occurred in obedience to natural law. No natural law was specially put in force for his destruction, and none suspended for his protection. All the chemistry and physics, in his case, were complete. Dr. Breed, according to his own showing, failed to interpret the phenomena rightly. From his published statement of the case, and in no unfriendly spirit, the results of my study of his case are presented in this memoir. As we differ so widely, it is not probable both are right, though, it is possible, both of us may be wrong. Through two decades of practice, I looked upon life and disease precisely as he did in this case, with the result of my being in a perpetual fog—no clear light in any direction. My philosophy was founded on conjecture, and my therapeutics on hope. But it is somewhat different now. Regarding the body as a unity, and disease, *per se*, a myth, the so-called diseases to which it is subject being regarded merely as *disarrangements*, and not as separate and special entities; and limiting my conceptions of the *modus operandi* of therapeutic, remedial, and hygienic agencies to their influence on the transformation of food into tissue, or constructive metamorphosis, and the oxidation or decay of tissues, so as to preserve its arrangements in the evolution of the forces of organic life; I certainly prescribe, as I believe, more intelligently, and it may be added, I think, more certainly and successfully than before. For myself, I have, in fact, as I fancy, in a great measure, transferred practical medicine into the domain of exact science. It was not done, however, without effort, and not accomplished at a single bound; for my progress was slow, labored, and gradual. I studied the steam engine, the magnetic telegraph, watches, the operations of the forces of inorganic nature around me, in all kinds of machinery, the correlation of the physical forces, and applied the methods of investigation in physical science to the elucidation of the phenomena of organic life, with the results stated. I am conscious that I am far from being perfect, have, indeed, a great deal to do, and but a fragment of life to do it in; but I have learned to observe and think on my legs, and in my carriage, as well as in my study; and so, when awake, am never altogether idle. The difficulties in the way of getting out of the established and well-worn "ruts" of thought and philosophy, great as they have been found, have all been surmounted. I do not any more think in the old channels. I ask this of all medicines, can you, or do you, aid in the transformation of food into flesh, or its waste or decay, by oxidation, so as to evolve normal, organic force, and the exit of the results of tissue decay properly from the body? If the empirical facts of therapeutics gives *no* for answer, then, I leave it with the apothecary, as of being no use to any of those who consult me for disarrangements of their bodies. The empirical facts of the *materia medica* suffice to obtain a tolerably clear conception of the actual or probable influence of any drug on constructive or destructive metamorphosis of the tissues. Nor is any serious difficulty encountered in reconciling the apparently contradictory facts of published therapeutics. For instance, the fixed alkalies, and most, if not all their combinations with acids, are agents to promote waste of tissue, and the elimination of the products of tissue metamorphosis out of the body. A memoir recently appeared in an Eastern journal, extolling the "bromide of potassium," in cholera infantum—a wasting disease (speaking in past ideas to me) of children, during the hot months of summer. Here is an apparent dilemma. "How can you reconcile that with your theory of the action of medicines?" asked a medical friend, who thinks my theory of life all stuff. In reply, I remarked to him, "why do children have the summer complaint?" "Why do they have frequent discharges from the bowels?" "and do those who perish generally wind up their lives in convulsions?" He replied to all the questions, "because they were exhausted by the hot weather." "Please to tell me what is exhaustion?" I asked, "why is it weakness?" he replied, "the warm weather exhausts them, and hence the bowel complaint." That is a fair sample of the muddle of life and therapeutics in which I was myself involved so many years. Now, my conception of the condition of things described as cholera infantum is, that, little by little, the tissues of children are constructed out of normal, molecular type, and, therefore, incapable of evolving normal organic dynamic force in their decay. To save the child, the "*vis meducatrix natura,*" the shadow of the laws of organic life, endeavors to remove this defective tissue by a peroxidation; the force evolved being, for the most part, correlated in heat. The debris of tissue metamorphosis, if retained in the body, wholly arrests the process of tissue repair. The bowel complaint is set up for the escape of these products from the body. If it is arrested by therapeutics, or otherwise; coma and convulsions speedily end the life of the patient. The bromide of potassium, by facilitating the oxidation of the defective tissues, acted in harmony with the laws of organic life, and was, therefore, eminently successful. But it is by no means the only agent or measure by which the same ends are accomplished. Chalk, mercury, blue pill, Rochelle salts, bitart potassa, castor oil, etc., all act in much the same way, *viz.*, by eliminating the debris of tissue metamorphosis, and promoting oxidation.

In conclusion, permit me to thank Dr. Breed, first, for reporting the case; and, secondly, for exposing the inner working of his mind in the study of it. It has afforded me a good apology for writing this memoir, for the sole purpose of aiding others in getting out of the muddle of phyisology, pathology, and therapeutics, in which I was so long enveloped myself, if there are any such—a very likely result, as, I think, in my own case, of the existing conceptions and philosophy of organic life.

